# Comparison of computed tomographic findings for radiolucent lesions of the mandibular ameloblastoma, odontogenic keratocyst, dentigerous cyst, and simple bone cyst

**DOI:** 10.1016/j.jds.2024.04.013

**Published:** 2024-04-25

**Authors:** Tomoki Sueyoshi, Junsei Sameshima, Naoki Kaneko, Toru Chikui, Hu Chen, Shiho Yokomizo, Haruki Nagano, Taiki Sakamoto, Shintaro Kawano

**Affiliations:** a Section of Oral and Maxillofacial Oncology, Division of Maxillofacial Diagnostic and Surgical Sciences, Faculty of Dental Science, Kyushu University, Fukuoka City, Fukuoka, Japan; b OBT Research Center, Faculty of Dental Science, Kyushu University, Fukuoka City, Fukuoka, Japan; c Department of Oral and Maxillofacial Radiology, Faculty of Dental Science, Kyushu University, Fukuoka City, Fukuoka, Japan

**Keywords:** Odontogenic keratocyst, Ameloblastoma, Dentigerous cyst, Simple bone cyst, Computed tomography, Diagnostic criteria

## Abstract

**Background/purpose:**

Radiolucent lesions of the mandible, including ameloblastoma, odontogenic keratocyst (OKC), dentigerous cyst (DC) and simple bone cyst (SBC), are frequently encountered in clinical practice. However, they vary in type and occasionally in appearance. Each lesion needs a different treatment and approach; therefore, accurate diagnosis is crucial before treatment. However, the radiographic findings, including computed tomography (CT), are often similar. This study aimed to compare the CT findings of 41 ameloblastomas, 74 OKCs, 87 DCs, and 13 SBCs in the mandible.

**Materials and methods:**

Patients were evaluated on initial CT images focusing on features such as long/short diameters, relationship with adjacent teeth, cortex appearance, locularity, scalloped margins, and sclerotic rims. Multivariate logistic regression analysis was performed to determine the most useful features for differential diagnosis. Criteria for differential diagnosis were finally established for three lesions, excluding SBC, which had a relatively small number of cases.

**Results:**

The relationship with the affected tooth, short diameters, sclerotic rims, and locularity were found to be significant features in the differential diagnosis. Ameloblastomas were characterized by relatively larger short diameters, frequently accompanied by root resorption and adjacent tooth displacement, while SBCs lacked these features. Sclerotic rims were commonly observed in OKCs and DCs, and most DCs were unilocular, containing a crown within the lesion. Based on these results, criteria were established for differential diagnosis with a diagnostic accuracy of 84.2%.

**Conclusion:**

This is the first study to attempt to characterize each lesion's radiological features, and these criteria are likely to assist in clinical diagnosis.

## Introduction

Radiolucent lesions of the mandible are commonly encountered in radiographs in clinical practice. Various diseases of the mandible can be depicted as radiolucent lesions, among which ameloblastomas, odontogenic keratocysts (OKCs), dentigerous cysts (DCs), and simple bone cysts (SBCs), which are characterized by radiolucent images, are particularly difficult to differentially diagnose compared to other diseases, despite their high frequency. OKC is a common cyst of odontogenic origin, whereas ameloblastoma is the most common odontogenic tumor.^1^, ^2^, ^3^, ^4^, ^5^, ^6^, ^7^, ^8^ DCs are the second most common type of cyst in the mandible, and solitary SBCs are also common. Differential diagnosis between these lesions is difficult because their clinical and radiographic features are so similar. They occur among patients within the same age distribution and most commonly appear in the posterior mandible, including the ramus and molar region.^9^^,^^10^ They may show some similar radiographic features, including unilocularity or multilocularity, association or no association with a tooth, or tooth displacement. Given the differences in their biological behavior and treatment as a result of the different characteristics of tumors, cysts, and pseudocysts, an accurate diagnosis before treatment is crucial.^11^, ^12^, ^13^

When radiolucent lesions are observed in dental/panoramic radiographs, computed tomography (CT) including cone beam computed tomography (CBCT) imaging is often performed to obtain more detailed information. Unlike other extraoral dental imaging procedures, CT acquires data volumetrically, providing three-dimensional radiographic imaging that reveals the relationship of the lesion to the surrounding tissues, as well as its internal characteristics, for assessing the dental and maxillofacial complex and facilitating dental diagnosis.^14^^,^^15^ Several different features in CT imaging have been proposed to differentiate between ameloblastoma, OKC, DC, and SBC.^16^^,^^17^ Although each lesion has several distinctive radiological features in the CT findings, no systematic statistical analysis has yet been undertaken to establish a differential diagnosis between these lesions. The present study involved relatively large-scale statistical analyses using the CT images of 41 patients with ameloblastoma, 74 patients with OKC, 87 patients with DC, and 13 patients with SBC in the mandible to reveal useful factors for differential diagnosis. This is the first study to compare CT features between the four lesions simultaneously. Finally, this study is the first to propose a model to help establish differential diagnostic criteria using the CT findings of radiolucent lesions, including ameloblastomas, OKCs, and DCs.

## Materials and methods

### Patients

This study included 41 patients with ameloblastoma, 74 patients with OKC, 87 patients with DC, and 13 patients with SBC who visited the Department of Oral and Maxillofacial Surgery, Kyushu University Hospital from 2007 to 2022. They were diagnosed with ameloblastoma, OKC, dentigerous cyst, or simple bone cyst by histopathological examination and CT or CBCT examination. Detailed information is shown in Table S1. This study excluded cases for which a definite diagnosis was not obtained by pathological biopsy and cases with artifacts that interfered with measurement in the slice sections requiring evaluation. The study design and methods were approved by the Institutional Review Board of the Center for Clinical and Translational Research of Kyushu University Hospital (IRB serial number: 22015-01). The methods were carried out in accordance with the approved guidelines.

### Methods

CT images were taken with the Aquilion 64-slice, Aquilion ONE (Toshiba Medical Systems Corporation, Tokyo, Japan). CBCT images were taken with 3D Accuitomo F17 (J. Morita MFG Corporation, Kyoto, Japan). Additionally, axial images (images scanned parallel to the Frankfort horizontal plane or mandibular inferior margin plane) and sagittal images (images scanned parallel to the midline of the mandible, dividing the mandible into left and right) were used in this study. The slice thickness was 2 or 4 mm. The region was divided into three parts (ramus, molar, or anterior) and defined as one of six types depending on the extent of the lesion (ramus, molar, anterior, ramus molar, molar anterior, and ramus molar anterior). To analyze the image of the lesion on the CT scans, nine items were measured following the method of Kawazu et al. and Kitisubkanchana et al. as shown in Table 1.^14^^,^^15^ The long diameter and short diameter were measured, as well as the largest diameter (long diameter) and the largest diameter perpendicular to the long diameter (short diameter) on the CT images where the transverse section of the lesion was largest (Fig. S1A). The bone cortex was examined for swelling, thinning, and loss on the buccal and lingual sides (Fig. S1B). The presence or absence of a scalloped margin was examined (Fig. S1C). The locularity was examined and divided into two groups: unilocular and multilocular (Fig. S1D). The presence or absence of a sclerotic rim was investigated (Fig. S1E). The relationship between the lesion and the impacted tooth was interrogated by classifying it into the following three groups according to where the lesion involved the tooth: the cemento-enamel junction (CEJ), the root, or the entire tooth (Fig. S1F).^14^ Tooth displacement and root resorption were recorded as yes or no (Figs. S1G and H). Sclerotic rim refers to lesions with a more diffuse zone of transition between the lesion and the normal surrounding bone.^16^ Three oral surgeons evaluated all CT images independently. In cases of disagreement between the observers, the images were re-examined, and a consensus evaluation was reached.

**Table 1:** Radiographic features examined in this study.

Long diameter
Short diameter
Cortex appearance
Locularity
Scalloped margin
Sclerotic rim
Relation between radiolucent lesion and impacted tooth
Adjacent tooth displacement
Root resorption

### Statistical analysis

GraphPad Prism version 10 (GraphPad Software, San Diego, CA, USA) and JMP software ver. 17 (JMP, Cary, NC, USA) were used for statistical analysis. The Kruskal–Wallis test was used to calculate *P* values for continuous non-parametric variables. The association between diagnosis and radiographic characteristics was analyzed using chi-squared tests. Multivariate logistic regression analysis was performed in two ways: one group of all four lesions and another group of ameloblastoma, OKC and DC. A *P* value of <0.05 was considered significant. The partition platform from JMP software ver. 17 was used for the differential diagnosis criteria. This platform recursively partitions data according to the relationship between explanatory and objective variables to create a decision tree. Its algorithm searches all possible branches of the explanatory variable to find the branch that most effectively predicts the response. The branches of the data are iterated, eventually forming a decision tree representing the rules for partitioning. Branching continues until a reasonable degree of fit is achieved. The algorithm selects the best fit from a large number of possible branches.

## Results

### Each lesion has a tendency for a specific region and characteristic size

**Figure 1:**
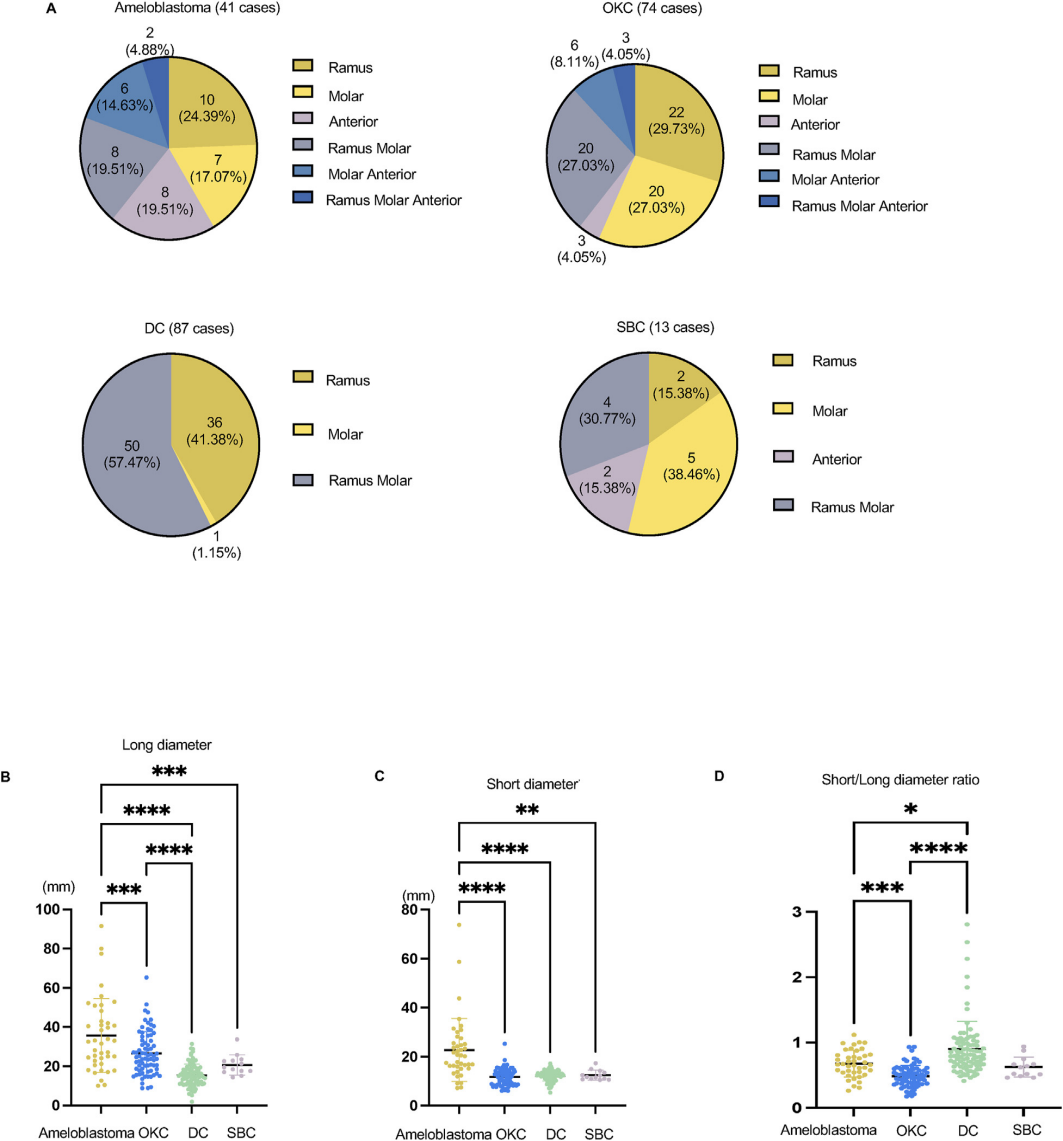
**Location and diameter in ameloblastoma, odontogenic keratocyst (OKC), dentigerous cyst (DC), and simple bone cyst (SBC).** (A) Circle charts showing site distribution of ameloblastoma, OKC, DC, and SBC. Ramus, molar, anterior, ramus molar, molar anterior, and ramus molar anterior regions are displayed in gold, yellow, purple, gray, indigo, and blue, respectively. (B–D) Dot plot data showing long diameter, short diameter, and short/long diameter ratio in ameloblastoma (gold), OKC (blue), DC (green), and SBC (purple). The Kruskal–Wallis test was used to calculate *P* values. Error bars represent mean ± SEM. ∗*P* < 0.05; ∗∗*P* < 0.01; ∗∗∗*P* < 0.001; ∗∗∗∗*P* < 0.0001.

The lesions were located mainly in the posterior regions in ameloblastoma, OKC, DC, and SBC (Fig. 1A). Ameloblastoma and SBC also occurred in the anterior area, which is relatively rare location for OKC. DC occurred only in the posterior area. The lesions were measured to investigate differences in size between ameloblastoma, OKC, DC, and SBC. The long and short diameters were distributed as shown in Fig. 1B and C. Both were longer in ameloblastoma than in OKC, DC, and SBC. Subsequent additional analysis revealed that the short/long diameter ratios were significantly higher in DC than in OKC and ameloblastoma (Fig. 1D).

### Changes in cortex appearance are standard features in all lesions

The presence of changes in cortex appearance on the buccal and lingual side was examined (Fig. S1B). The results are shown in Table 2. Buccal expansion, thinning, and disappearance were prominent in ameloblastoma, and buccal thinning was also prominent in OKC and SBC. There was a significant difference in buccal cortex appearance. Lingual thinning was observed in all lesions, while lingual disappearance was frequently observed in ameloblastoma. In general, changes in both buccal and lingual cortex appearance was a frequent feature in all lesions.

**Table 2:** Cortex appearance.

		Buccal				*P* value
		Ameloblastoma	OKC	DC	SBC	
Expansion	+	35	41	27	8	*P* < 0.0001
	–	6	33	60	5	
Thinning	+	38	66	62	12	*P* < 0.01
	–	3	8	25	1	
Disappearance	+	26	23	9	1	*P* < 0.0001
	–	15	51	78	12	
		Lingual				*P* value
		Ameloblastoma	OKC	DC	SBC	
Expansion	+	33	48	50	11	*P* < 0.05
	–	8	26	37	2	
Thinning	+	40	71	77	13	n.s.
	–	1	3	10	0	
Disappearance	+	29	26	26	2	*P* < 0.0001
	–	12	48	61	11	

OKC: Odontogenic keratocyst.

DC: Dentigerous cyst.

SBC: Simple bone cyst.

Pearson chi-squared test.

### OKC and DC tend to be unilocular and ameloblastoma features a sclerotic rim less frequently than the other three lesions

A scalloped margin was present less frequently in DC (11.5%) than in the other three lesions (Table 3). Both OKC and ameloblastoma are known to be unilocular or multilocular in appearance, and DCs often have a unilocular appearance on CT images (Fig. S1D). The CT images of all lesions were examined for septal structures. Multilocular lesions were observed in 26 of 41 cases (63.4%) of ameloblastoma and 22 of 74 cases (29.7%) of OKC (Table 3). Additionally, 84 of 87 cases (96.6%) of DC were unilocular. In SBC, the frequency of unilocularity and multilocularity was approximately comparable. A sclerotic rim was frequently observed between the lesions and the surrounding bone in the three lesions excluding ameloblastoma, and there was a significant difference.

**Table 3:** Presence of scalloped margin, locularity, and sclerotic rim.

		Ameloblastoma	OKC	DC	SBC	*P* value
Scalloped margin	+	25	36	10	10	*P* < 0.0001
	–	16	38	77	3	
Locularity	Unilocular	15	52	84	7	*P* < 0.0001
	Multilocular	26	22	3	6	
Sclerotic rim	+	10	44	69	11	*P* < 0.0001
	–	31	30	18	2	

OKC: Odontogenic keratocyst.

DC: Dentigerous cyst.

SBC: Simple bone cyst.

Pearson chi-squared test.

### Adjacent tooth displacement is significantly abundant in ameloblastoma and DC, and root resorption is frequently observed in ameloblastoma

The relationship to adjacent teeth is an important finding in diagnosing radiolucent lesions in the mandible. DC involves the crown of the impacted tooth within a cystic cavity: the typical finding is thus the cyst wall rising from the CEJ or root. Indeed, the DC lesion surrounds the tooth and is attached to the CEJ or the root beyond the CEJ in most cases (Table 4). Displacement of adjacent teeth was frequently observed in DC (87.3%) and ameloblastoma (70.7%) (Table 5). Root resorption was frequently observed in ameloblastoma (48.8%), but seldom in either OKC (23.0%) or DC (18.4%) (Table 5). Notably, adjacent tooth displacement and root resorption were completely absent in SBC.

**Table 4:** Relationship between radiolucent lesion and impacted tooth.

		Ameloblastoma	OKC	DC	SBC
Relation between radiolucent lesion and impacted tooth	CEJ	3	8	62	1
	Root	2	9	23	0
	Entire tooth	5	10	2	0
	None	31	47	0	12

OKC: Odontogenic keratocyst.

DC: Dentigerous cyst.

SBC: Simple bone cyst.

CEJ: cemento-enamel junction.

**Table 5:** Presence of adjacent tooth displacement and root resorption.

		Ameloblastoma	OKC	DC	SBC	*P* value
Adjacent tooth displacement	+	29	30	76	0	*P* < 0.0001
	–	12	44	11	13	
Root resorption	+	20	17	16	0	*P* < 0.0001
	–	21	57	71	13	

OKC: Odontogenic keratocyst.

DC: Dentigerous cyst.

SBC: Simple bone cyst.

Pearson chi-squared test.

### Multivariate analysis reveals significant features and establishes the criteria for differential diagnosis

Multivariate analysis was used to determine statistical links between variables and extracted diagnostic predictors. Because the number of SBC cases was relatively small, multivariate logistic regression analysis was performed in two ways: one group of all four lesions and another group consisting of ameloblastoma, OKC, and DC (Table 6, Table 7). The relationship between the radiolucent lesion and the impacted tooth, short/long diameter ratio, short diameter, sclerotic rim, and locularity were significant factors common to the two groups.

**Table 6:** Multiple regression analysis between ameloblastoma, OKC, DC, and SBC.

Ameloblastoma, OKC, DC and SBC		
Variate	Wald χ	*P* value
Relation between radiolucent lesion and impacted tooth	277.75	<0.0001
Short/long diameter ratio	42.37	<0.0001
Short diameter	28.00	<0.0001
Scalloped margin	19.77	0.0014
Screlotic rim	10.09	0.0015
Locularity	13.31	0.0040
Adjacent tooth displacement	3.33	0.1883
Lingual expansion	1.61	0.2041
Buccal expansion	0.12	0.7226
Buccal disappearance	0.002	0.9628
Long diameter	0	1.0000
Buccal thinning	0	1.0000
Lingual thinning	0	1.0000
Lingual disappearance	0	1.0000
Root resorption	0	1.0000

OKC: Odontogenic keratocyst.

DC: Dentigerous cyst.

SBC: Simple bone cyst.

**Table 7:** Multiple regression analysis between ameloblastoma, OKC, and DC.

Ameloblastoma, OKC and DC		
Variate	Wald χ	*P* value
Relation between radiolucent lesion and impacted tooth	166.84	<0.0001
Short/long diameter ratio	14.24	0.0008
Short diameter	10.80	0.0010
Sclerotic rim	11.80	0.0027
Locularity	11.41	0.0097
Adjacent tooth displacement	5.94	0.0148
Lingual expansion	2.16	0.1416
Buccal thinning	1.21	0.2709
Lingual disappearance	0.76	0.3810
Buccal expansion	0.71	0.3994
Root resorption	0.15	0.6965
Scalloped margin	0.66	0.7170
Buccal disappearance	0.05	0.8128
Long diameter	0.22	0.8917
Lingual thinning	0	1.0000

OKC: Odontogenic keratocyst.

DC: Dentigerous cyst.

SBC: Simple bone cyst.

**Figure 2:**
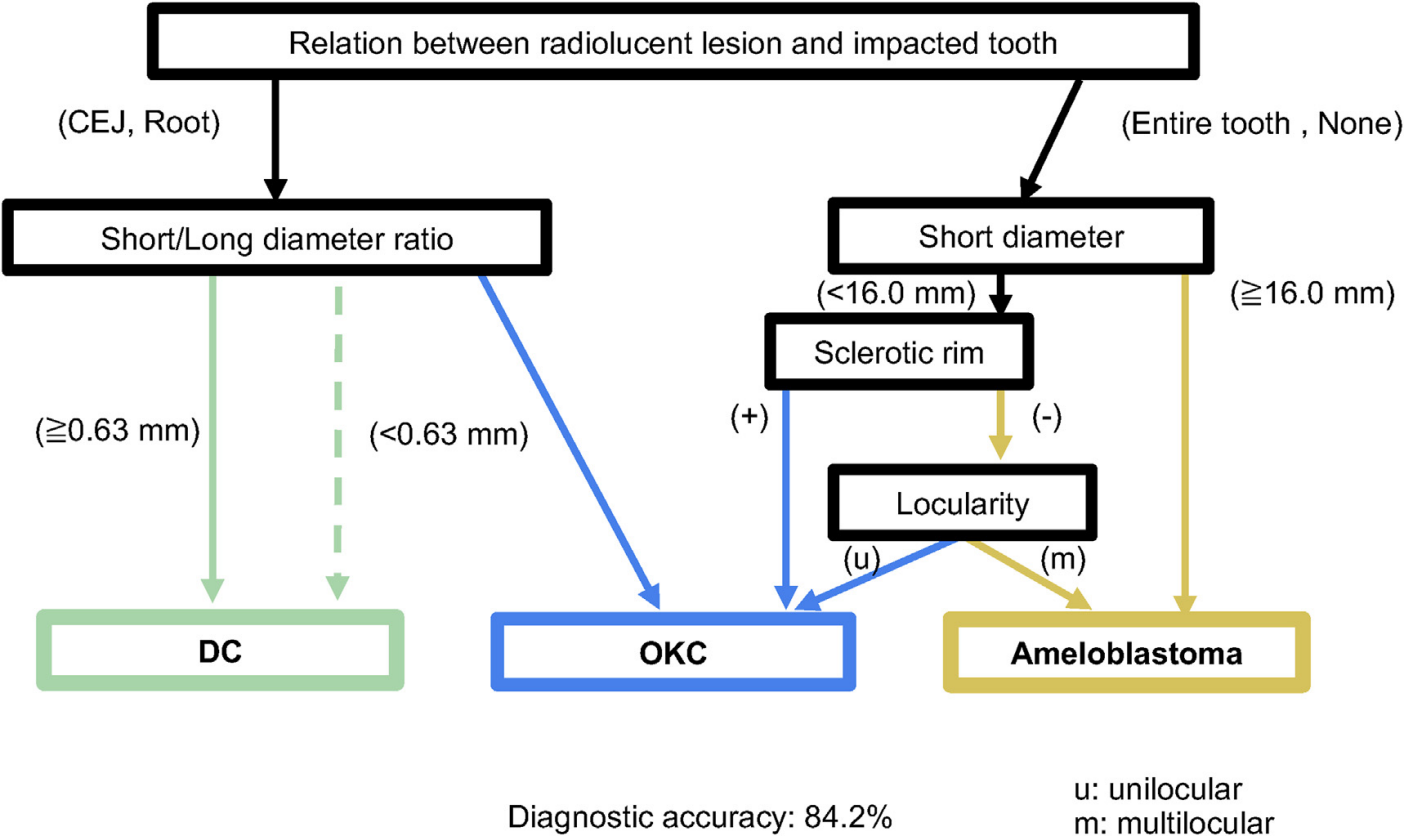
**Criteria for differential diagnosis.** The relationship between the radiolucent lesion and the impacted tooth is used first for differential diagnosis. Cases are separated into cemento-enamel junction (CEJ) or Root and Entire tooth or None. The long/short diameter ratio, short diameter, sclerotic rim, and locularity are also included. Cases with a long/short diameter ratio of ≥0.63 are predicted to be odontogenic keratocysts (OKC). Cases with a long/short diameter ratio of <0.63 are predicted to be dentigerous cysts (DC), while a certain number of OKC cases are included (dashed line). The presence of a sclerotic rim predicts OKC in cases with a short diameter of <16.0 mm, while unilocularity predicts OKC in cases without a sclerotic rim. Ameloblastoma is predicted in cases with a short diameter of ≥16.0 mm. Cases with a short diameter of <16 mm but no sclerotic rim and multilocularity are predicted to be ameloblastoma. All cases are ultimately divided into ameloblastoma, OKC, and DC as a predicted diagnosis with 84.2% diagnostic accuracy.

The criteria for differential diagnosis were finally established for three lesions, excluding SBC, which had a relatively small number of cases. The relationship between the radiolucent lesion and impacted tooth, short/long diameter ratio, short diameter, sclerotic rim, and locularity were selected as explanatory variables for differentiation. These factors also showed significant differences in the multivariate analysis. The criteria are summarized in Fig. 2. The diagnostic accuracy rate for these criteria was 84.2% (Table 8).

**Table 8:** Comparison of predicted and histopathological diagnosis.

Histopathological diagnosis	Predicted diagnosis			Total
	OKC	Ameloblastoma	DC	
OKC	65	8	1	74
Ameloblastoma	1	35	5	41
Dentigerous cyst	17	0	70	87
Total	83	43	76	202

OKC: Odontogenic keratocyst.

DC: Dentigerous cyst.

## Discussion

In a previous study, we showed that short diameter and locularity were significant features to aid in differentiation between ameloblastoma and OKC by multivariate logistic regression analysis using a smaller number of samples than the present study.^18^ However, the association with teeth was not investigated, and DC and SBC, which are frequently encountered in clinical practice, were omitted. To date, no systematic study has compared the radiological features of ameloblastoma, OKC, DC, and SBC. This is the first study to attempt to characterize the radiological features of each lesion by investigating a relatively large number of cases.

The critical feature in separating ameloblastoma or DC was the relationship between the radiolucent lesion and the impacted tooth, which occupies the first position in the decision tree. The relationship between the radiolucent lesion and the impacted tooth may reflect the origin of the lesion. DC is considered a cystification of the odontogenic epithelium after the crown has finished forming; thus, the cyst wall arises from the CEJ or the side of the root. Ameloblastoma and OKC begin in the cells forming the protective enamel lining and hard tissue of normal teeth before tooth development, respectively. Similarly, the origin of SBC is unrelated to tooth development. In other words, in ameloblastoma, OKC and SBC, the cyst wall is unlikely to develop from the CEJ or root, although it may not contain the tooth within the lesion or may contain the entire tooth as a result of growth of the lesion.

The short diameter and short/long diameter ratio, which are used to discriminate between OKC and ameloblastoma or OKC and DC, may reflect the growth pattern of the lesion. In our previous study comparing ameloblastoma and OKC, the short diameter was included as a significant feature, and 16.0 mm was calculated as the cut-off value that separates ameloblastoma from the other lesions. In a previous mandibular morphology study using quantitation of CT images, the mean buccolingual width was reported as 16.1 mm for males and 15.1 mm for females at the thickest part of the second molar distal section and the buccal cortex is thicker than the lingual cortex.^19^^,^^20^ These previous reports indicate that a short diameter of >16.0 mm suggests the presence of buccal expansion. Similarly, a small short/long diameter ratio suggests the tunneling-type growth pattern observed in OKC.

Locularity was also an essential factor in differential diagnosis in this study. As can be inferred from the origin of the lesion, most DCs are unilocular, but there are rare cases with thin septa.^21^ SBC occasionally exhibited multilocularity because of the propensity of the lesion to scallop the inside of the outer cortex of the mandible.^22^ Our results were consistent with previous reports, but it is noteworthy that OKC is frequently unilocular, even if its short diameter and size are relatively large. Our decision tree includes the presence of a sclerotic rim as an aid in differentiating OKC from ameloblastoma. This radiopaque rim represents reactive bone and may indicate the lesion's ability to stimulate surrounding bone production.^23^ Ameloblastoma showed a tendency for the absence of a sclerotic rim, while OKC exhibited the opposite tendency, suggesting that the presence of a rim depends on the aggressiveness of the neoplasm and the lesion's growth pattern.^16^

Criteria were finally created based on multivariate logistic regression analysis, and the diagnostic accuracy rate was found to be 84.2%. This rate indicates the possibility of clinical application with some validity. However, as indicated by the dashed line within the criteria, a condition that contributes significantly to the decline in accuracy was also evident: cases with cyst walls arising from the CEJ and root with a small short/long diameter ratio turn out to be a mixture of OKC and DC. These results indicate that OKC can incidentally contain the CEJ and roots in accordance with the growth of the lesions, and that DCs may also exhibit a tunneling-type growth pattern similar to that of OKC when the cortical bone is thicker. Although there is room for further improvement in our criteria, and clinical diagnosis should be based on numerous clinical findings, our criteria may be applied as a diagnostic aid.

In summary, valuable features for the differential diagnosis of radiolucent lesions of the mandible were identified by comparing and analyzing CT findings. The relationship to the impacted tooth, short diameter, short/long diameter ratio, and the presence of a sclerotic rim were found to be significant features to aid in differentiation between ameloblastoma, OKC, DC, and SBC; these criteria may be applicable to clinical practice. However, our study had several limitations. First, SBC has not been included in the criteria, and the subtypes of ameloblastoma have not been included due to the relatively small number of cases. In addition, maxillary lesions present a more varied CT image due to adjacent structures, such as the nasal cavity and maxillary sinus, which were not investigated in this study for the same reason. Second, the study investigated only plain CT imaging without a contrast agent. It is still debatable how useful contrast imaging and magnetic resonance imaging (MRI) are for diagnosing these lesions, but a comprehensive approach is needed to improve diagnostic accuracy.^24^^,^^25^ Further studies assessing large samples and including enhanced CT, MRI, and other similar lesions in both mandible and maxilla are required to allow better diagnosis as well as to differentiate subtypes.

## Declaration of competing interest

The authors have no conflicts of interest relevant to this article.
