# Supplementary figures and images for: Comparison of computed tomographic findings for radiolucent lesions of the mandibular ameloblastoma, odontogenic keratocyst, dentigerous cyst, and simple bone cyst
Source: J Dent Sci. 2024 Apr 25;20(1):605–12. doi: 10.1016/j.jds.2024.04.013 (PMC11762212; doi:10.1016/j.jds.2024.04.013)

Supplementary Figure 1

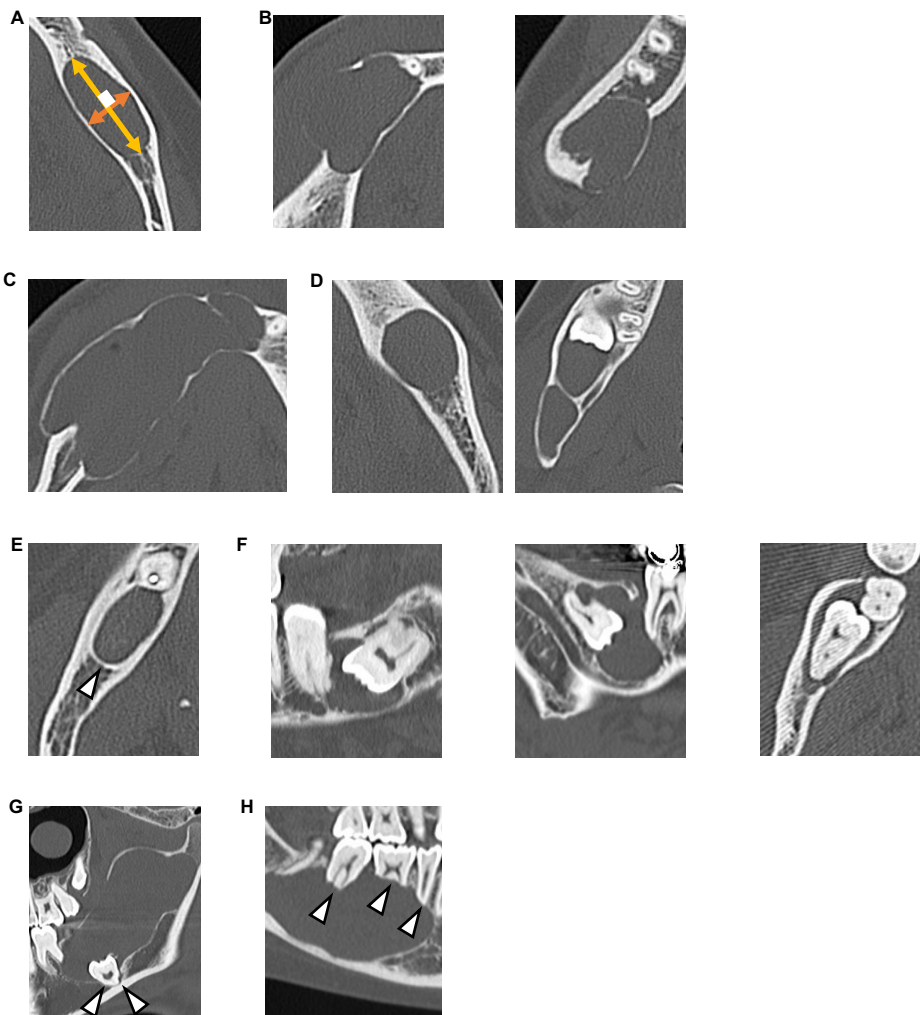

Supplement: Multimedia component 1 [file mmc1.pdf]
