# Supplementary material for: Comparison of computed tomographic findings for radiolucent lesions of the mandibular ameloblastoma, odontogenic keratocyst, dentigerous cyst, and simple bone cyst
Source: J Dent Sci. 2024 Apr 25;20(1):605–12. doi: 10.1016/j.jds.2024.04.013 (PMC11762212; doi:10.1016/j.jds.2024.04.013)
Supplement: Multimedia component 2 [file mmc2.docx]

**Table S1**. Subjects

|  |  | Ameloblastoma | OKC | DC | SBC |
| --- | --- | --- | --- | --- | --- |
| Number of cases |  | 41 | 74 | 87 | 13 |
| Sex | Male | 25 | 47 | 52 | 3 |
|  | Female | 16 | 27 | 35 | 10 |
| Mean age |  | 40.8±19.1 | 44.9±19.9 | 45.3±13.2 | 42.5±20.6 |
| Range of ages  (years) |  | 8-81 | 11-87 | 12-75 | 12-71 |

OKC: Odontogenic keratocyst

DC: Dentigerous cyst

SBC: Simple bone cyst

**Supplementary figure legends**

**Figure S1.** Representative images of each radiographic feature

(A) The image shows how to measure long and short diameters in the largest transverse section of the lesion. (B) Representative images of bone expansion, thinning, and disappearance. (C) Scalloped margin. (D) Unilocular (left) and multilocular (right) structures. (E) Sclerotic rim in odontogenic keratocyst (OKC) (white arrowhead). (F) Relationship between radiolucent lesion and impacted tooth (cemento-enamel junction [CEJ], Root, Entire tooth and None). (G) Adjacent tooth displacement (white arrowhead). (H) Root resorption (white arrowhead).
